# Procalcitonin as prognostic marker in severe sepsis of abdominal origin

**DOI:** 10.1186/cc14138

**Published:** 2015-03-16

**Authors:** A Gonzalez-Lisorge, C Garcia-Palenciano, G Ercole, T Sansano-Sanchez, M Campos Aranda, F Acosta Villegas

**Affiliations:** 1 Hospital Universitario Virgen de la Arrixaca, Murcia, Spain; 2 Universidad de Murcia, Murcia, Spain

## Introduction

We evaluated the utility of procalcitonin (PCT) as a marker of outcome in severe sepsis of abdominal origin (SSAO). SSAO is one of the most prevalent pathologies in surgical ICUs (sICUs). Mortality from 19 to 70% has been reported. Biomarkers are basic tools for diagnosis, follow-up and outcome of sepsis. One of the most studied in last decades has been PCT. Its levels and kinetics could be useful to evaluate the outcome of septic patients.

## Methods

We studied all patients admitted to a sICU with SSAO, from 2007 to 2008. Data collected were: PCT levels on days 1, 3 and 7, gender, age, APACHE II on admission, positivity of surgical cultures, microorganisms isolated and sepsis origin (community or nosocomial).

## Results

Sixty-nine patients were included. Mortality was 23%. Median age was 64.94 years. Median APACHE II was 16.43 points. At day 1, PCT levels were higher in survivors (S) than in exitus (E) (S: 29.22 ng/ ml vs. E: 14.93 ng/ml, *P *< 0.05). PCT levels were influenced by gender (males: 27.74 ng/ml vs. females: 15.04 ng/ml, *P *< 0.05), positive cultures (positive: 25.25 ng/ml vs. negative: 13.49 ng/ml, *P *< 0.05) and isolation of Gram-negative microorganisms (Gram-negative: 27.53 ng/ml vs. Gram-positive: 14.77 ng/ml, *P *< 0.05). Patients with community-acquired sepsis had higher levels of PCT on admission (37.53 ng/ml vs. 13.29 ng/ml, *P *< 0.02). None of these factors had an influence on mortality. On day 3 PCT levels where higher in S (S: 20.65 ng/ml vs. E: 16.23 ng/ml, *P *< 0.05). On day 7 PCT levels were higher in E (S: 3.54 ng/ ml vs. E: 12.88 ng/ml, *P *< 0.05). PCT kinetics was different depending on outcome. E patients presented persistently higher levels, whereas PCT in S decreased over time (*P *< 0.05). PCT on day 7 best identified outcome (AUC ROC 0.768). PCT ≥3.5 ng/ml predicted mortality (sensitivity 55%, specificity 73%).

## Conclusion

PCT on day 7 and PCT kinetics can be useful to predict outcome in SSAO. PCT is higher in community-acquired sepsis, when surgical cultures are positive, in Gram-negative isolations and in males.
